# Specific Bile Microorganisms Caused by Intra-Abdominal Abscess on Pancreaticoduodenectomy Patients: A Retrospective Cohort Study

**DOI:** 10.3390/curroncol29010009

**Published:** 2021-12-27

**Authors:** Young-Jen Lin, Te-Wei Ho, Chien-Hui Wu, Ting-Chun Kuo, Ching-Yao Yang, Jin-Ming Wu, Yu-Wen Tien

**Affiliations:** 1 Department of Surgery, National Taiwan University Hospital, National Taiwan University College of Medicine, Taipei 100, Taiwan; young332@ntuh.gov.tw (Y.-J.L.); 008835@ntuh.gov.tw (T.-W.H.); b91401112@ntu.edu.tw (C.-H.W.); tina@ntuh.gov.tw (T.-C.K.); cyang@ntuh.gov.tw (C.-Y.Y.); 2 Department of Surgery, National Taiwan University Hospital Hsin-Chu Branch, Hsin-Chu County 302, Taiwan

**Keywords:** pancreaticoduodenectomy, biliary drainage, positive bile culture, intra-abdominal abscess, surgical complication

## Abstract

We retrospectively collected PD patients with a performance of bile culture between 2007 and 2019 in our institute. As to bile culture, we used a swab to do intraoperative bile cultures after transection of the CBD. IAA was defined as the documental bacteriological culture from either a turbid discharge from the intraoperatively placed drain in patients with a clinical picture consistent with infection or a postoperative fluid collection managed by CT-guided placement of drains. A total of 1244 PD patients were identified, and 539 (43.3%) subjects with bile sampling were included for analysis. Among these study patients, 433 (80.3%) developed bile contamination (positive bile culture). Bile contamination showed a significantly higher rate of IAA compared to non-bile contamination (17.1% vs. 0.9%, *p* < 0.001). The rate of co-shared microorganisms in both bile and abscess was 64.1%. On the multivariate analysis, age and specific bile microorganisms (Enterococcus species, Escherichia Coli, Streptococcus species, Citrobacter species, and Candida) are significantly associated with development of IAA. Specific bile microorganisms are the highly significant factors associated with development of IAA. The strategy to prevent bile spillage during PD should be considered to minimize afterward contamination of the abdominal cavity and prevent IAA.

## 1. Introduction

Pancreaticoduodenectomy (PD) is one of the most complex abdominal operations, accompanied by a considerable rate of postoperative complications and mortality. Despite advances in surgical techniques, perioperative care, and a trend toward a decreasing rate of surgical death, most high-volume centers still report a steadily high rate of postoperative morbidity (ranged from 30% to 60%) [1,2,3]. Among the PD-associated complications, intra-abdominal abscess (IAA) is the leading cause of surgical mortality in PD cases that warrants prompt antibiotic treatment, prolonged hospital stays, or interventional drainage; otherwise, a sepsis/septic shock could occur, posing a danger to patients’ lives [4,5,6]. Work to investigate the risk of IAA is very important to improve surgical quality on PD patients.

Obstructive jaundice is the most common symptom in PD patients with periampullary cancer [7]. Although pre-operative biliary drainage (PBD), including percutaneous transhepatic cholangiography and drainage (PTCD) or endoscopic retrograde biliary drainage (ERBD) [8], may be necessary in selected PD cases preoperatively to relieve hyperbilirubinemia and to control cholangitis by biliary stents, which increases the rates of complications [9]. Notably, recent studies supported the cause–effect relationship between PBD and IAA [10,11], whereas the mechanism was unknown.

In this study, we asked whether bile contamination (positive bile culture) in PD patients will cause IAA. Our hypothesis is that the microorganisms in the bile will spread over the abdominal cavity after transection of proximal CBD and develop the abscess afterward. The aim of this study was to clarify the effect of bile contamination in the development of IAA and to characterize the infectious pathogens between bile and the abscess.

## 2. Materials and Methods

This single-center prospective study was approved by the Ethics Committee, and the work has been reported in line with the STROCSS criteria [12]. In this study, the patients without bile sampling were excluded. Periampullary cancer was defined below according to the International Classification of Diseases, 9th Revision, Clinical Modification (ICD-9-CM) and pathological report: pancreatic ductal adenocarcinoma (ICD-9-CM code 157.0), distal common bile duct cancer (ICD-9-CM code 156.1), ampulla of Vater cancer (ICD-9-CM code 156.2), and duodenal cancer (ICD-9-CM code 152.0). Biliary drainage was achieved by endoscopic placement of either a plastic or metal stent. The choice of PD procedure (classical Whipple procedure or pylorus-preserving Whipple procedure) depended on the extension of the tumor, as previously described [11]. The postoperative pancreatic fistula (POPF) was defined according to the 2016 International Study Group of Pancreatic Surgery [13], and only clinically relevant fistulae were included in the analysis.

### 2.1. Antimicrobial Prophylaxis

Antibiotic prophylaxis consisted of cefmetazole 1 g i.v., both before PD and repeated every 3 h during the operation. In cases of allergy, intravenous ciprofloxacin and metronidazole were used. On post-operative course, we kept the use of antibiotics for at least five days, which were either discontinued in non-infectious cases or tailored for patients experiencing major infectious post-operative complications according to the antibiotic sensitivity of sampling bile or abscess cultures.

### 2.2. Bile Cultures and Microbiology

Bile was obtained routinely by the senior author (Y.W.T.) and, at the surgeons’ discretion, by the other authors depending if the infected bile was observed. As to bile culture, we used a swab to do intraoperative bile cultures after transection of the CBD. The swab was kept at room temperature and delivered to the microbiology laboratory within two hours of collection. Afterward, cultures were performed aerobically and anaerobically according to standard laboratory protocols and assessment of the antibiotic resistance pattern in patients with bile contamination [14]. A positive bile culture was defined as the swab growing microorganisms.

Further, we would analyze the effect of antimicrobial medication on IAA. Ineffective therapy was defined as bile microorganisms treated with an inappropriate antimicrobial drug (ID) or drug resistance (DR). ID was defined as an antimicrobial regimen that does not address the underlying causative organism. Furthermore, the definition of DR was based on minimal inhibitory concentration breakpoints [15].

### 2.3. Intra-Abdominal Abscess & Mortality

Data on complications included all complications following surgery until discharge and/or readmission [16]. IAA was defined as the documental bacteriological culture from either a turbid discharge from the intraoperatively placed drain in patients with a clinical picture consistent with infection or a postoperative fluid collection managed by CT-guided placement of drains. Microorganisms isolated from postoperative samples (cultures from intra-abdominal abscess) were compared with those from intraoperative bile samples. Postoperative mortality included all event and deaths occurring within 30 days of operation and duration of hospital stay.

### 2.4. Statistical Analysis

Statistical analyses were performed utilizing the SPSS version 23 (IBM, Armonk, NY, USA). Data are presented as median (interquartile range [IQR]), odds ratio (OR) (95% CI), or number (percentage). Categorical variables were compared using the chi-squared test, or Fisher exact test if the numbers were below 5. Continuous data were compared using the nonparametric Mann–Whitney U test, and some continuous variables were dichotomized according to predetermined thresholds, which were analyzed as categorical variables. The potential covariates associated with development of IAA were included further in a multivariate analysis via the binary logistic regression model. All statistics were two-tailed, and differences were considered significant when *p* < 0.05.

## 3. Results

There were 1244 consecutive PD cases from 2007 to 2019. In this study, we excluded the patients without conducting bile cultures (*n* = 705). Among the 539 subjects with bile sampling, there were 433 with bile contamination. Among the study population with bile contamination (N = 433), 184 (42.5%) cases had ineffective therapy, including 130 ID and 54 DR. Of the 130 cases with ID, Enterococcus species and Candida were the most common microorganisms, on which the second-generation cephalosporin had no antimicrobial effects.

Further, we divided the study population into two groups according to the status of bile contamination (Table 1). The bile contamination group was more likely to be older (66.4 ± 8.5 vs. 60.8 ± 8.9 years; *p* = 0.006), with higher maximal value of serum bilirubin both at any time (2.6 vs. 0.9 mg/dl; *p* < 0.001) and within two days before surgery (1.4 vs. 0.9 mg/dl; *p* = 0.001), receiving antibiotics within 30 days before surgery (70.0% vs. 50.0%; *p* < 0.001), receiving preoperative biliary drainage (41.8% vs. 19.8%; *p* < 0.001), with a longer operation time (260 ± 42.5 vs. 223 ± 37.0; *p* <0.001) and higher intra-abdominal abscess rate (17.1% vs. 0.9%; *p* < 0.001) than the no bile contamination group. There were seven mortalities in this cohort. There was no statistical difference in the occurrence of the clinically relevant pancreatic fistula (25.4% vs. 26.4%; *p* = 0.610) between the bile contamination group and no bile contamination group. The cause of mortality was IAA-related septic shock in five patients (71.4%), hepatic failure in one patient (14.3%), and myocardial infarction in one patient (14.3%).

**Figure 1:**
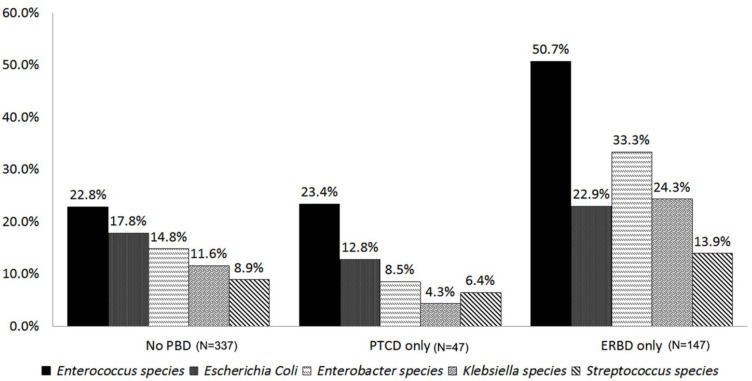
Comparison of microorganisms grown in intraoperative bile cultures between different PBD.

**Figure 2:**
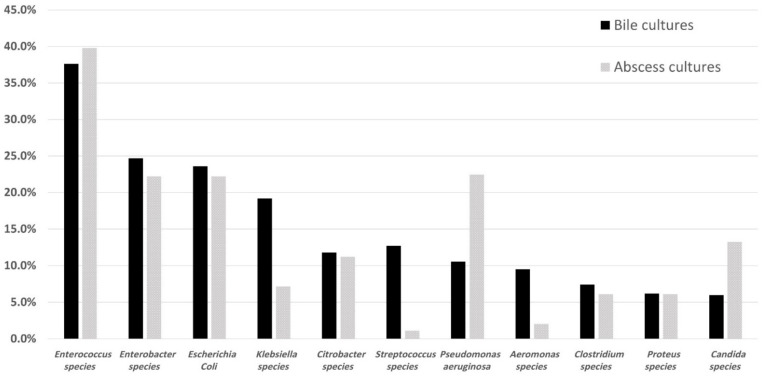
Comparison of microorganisms grown in intraoperative bile cultures and intra-abdominal abscess cultures.

Further, the microorganisms cultured in the swab were analyzed. The predominant microorganisms were Enterococcus species (163 cultures [37.6%]), Enterobacter species (107 cultures [24.7%]), Escherichia coli (102 cultures [23.6%]), and Klebsiella species (83 cultures [19.2%]). The distribution of bile microorganisms grouped by preoperative biliary drainage was shown in Figure 1. The pattern between non-PBD and PTCD was similar, whereas ERBD was relatively different from them. Furthermore, we compared the pattern of the microorganisms grown in intraoperative bile cultures and IAA cultures (Figure 2). Both Enterococcus and Enterobacter cloacae are the most common co-cultured organisms. Among the 248 subjects with simultaneous positive bile culture and abscess cultures, 159 patients (64.1%) had co-shared microorganisms.

We enrolled the top ten most common microorganisms and Candida species in bile sampling into a regression analysis to predict the development of IAA (Table 2). The Candida species (OR = 4.994; 95% CI: 1.837–13.572; *p* = 0.002), Enterococcus species (OR = 4.156; 95% CI: 2.661–6.491; *p* < 0.001), Citrobacter species (OR = 3.376; 95% CI: 1.686–6.762; *p* = 0.001), Streptococcus species (OR = 2.796; 95% CI: 1.502–5.206; *p* = 0.001), Enterobacter species (OR = 2.515; 95% CI: 1.546–4.093; *p* < 0.001), and Escherichia coli (OR = 2.333; 95% CI: 1.837–13.572; *p* = 0.002) are significantly associated with IAA. Lastly, we enrolled these six pathogens into the final regression model (Table 3). It showed that age (OR = 1.023; 95% CI: 1.004–1.041; *p* = 0.014) and all of the six pathogens including Candida species (OR = 4.666; 95% CI: 1.677–12.981; *p* = 0.003), Enterococcus species (OR = 4.103; 95% CI: 2.609–6.454; *p* < 0.001), Citrobacter species (OR = 3.050; 95% CI: 1.510–6.159; *p* = 0.002), Enterobacter species (OR = 2.231; 95% CI: 1.349–3.689; *p* = 0.002), Streptococcus species (OR = 2.519; 95% CI: 1.331–4.767; *p* = 0.005), and Escherichia coli (OR = 2.215; 95% CI: 1.336–3.671; *p* = 0.002) are significantly associated with the development of IAA.

To analyze the effect of ineffective IAA, an adjusted model was created (Table S1). It showed that ineffective therapy was significantly associated with IAA (OR = 2.725; 95% CI: 1.840–4.036; *p* < 0.001).

## 4. Discussion

IAA is one of the most serious surgical infectious complications that may pose deleterious consequences [17,18,19], and it requires antibiotic treatment, wound debridement, or interventional drainage [4]. The present study showed that more than 70% of the mortality was associated with IAA-related septic shock and demonstrated that specific bile microorganisms were the most significant risk factor contributing to development of IAA.

**Figure 3:**
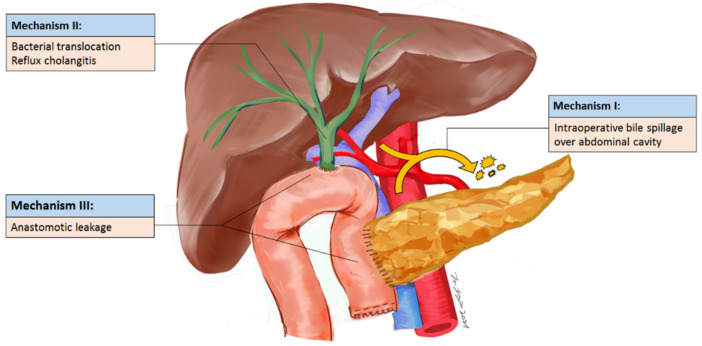
Potential mechanism of intra-abdominal abscess.

To the best of our knowledge, this is one of the largest studies to address the association between bile contamination and IAA, because bile culture is not routinely performed in PD patients and is not considered as an important factor for clinical outcomes. Recently, several studies challenged the role of PBD because it was associated with higher rates of surgical infectious complications and mortality (up to 9.8%) [9,20,21,22]. However, the mechanism has not been well investigated. The patients with PBD had higher proportions of bile contamination due to both insertion of biliary stents inside CBD and frequent reflux cholangitis (sphincterotomy performed during ERBD). The results of this study show that specific bile microorganisms are the highly significant factor associated with IAA. The proximal CBD would be left unclamped before choledochojejunostomy to prevent prolonged cholestasis [23], so bile contamination may migrate from the bile duct to the abdominal cavity or through the fascia to the subcutaneous tissue. This hypothesis was also supported by the phenomenon that more than half of the patients with simultaneously positive bile and abscess cultures had the co-cultured microorganisms. The above reasons may partially explain why bile contamination resulted in IAA or other surgical infectious complications, and positive bile culture may be the “upstream” cause of surgical infectious complications (Figure 3). Notably, there are around 36% discordant cultures between bile and abscess. In addition to our proposed mechanism, there are two other possible mechanisms for IAA, including bacterial translocation in the small bowel and anastomotic leakages. Microorganisms in the small bowel may cause IAA afterward, explaining the discordant cultures between bile and IAA.

Our study was similar to previous literature that Enterococcus species and Escherichia coli, as the predominant microorganism and common pathogens isolated from bile and abscess, shared co-cultured incidences up to 49% [24]. Some studies addressed the correlation between bile contamination and surgical site infections [8,25,26]. Moreover, specific bile microorganisms, such as Escherichia coli [1] or Enterococcus faecium/Enterobacter cloacae [14,25], were associated with higher rates of infectious complications. Several reports address that Klebsiella colonization is associated with infectious complications [27,28]. In our study, this correlation was not significant. The main reason to explain the inconsistent findings may be the selection of prophylactic antibiotics. We routinely used cefmetazole, which had the antimicrobial coverage of Klebsiella species, in contrast to first-generation cephalosporin. In addition to bacterial pathogens, some articles addressed that Candida species in bile were a significant factor in developing IAA [29,30]. It was considered that the fungal infection process not only caused physical damage to organ walls, allowing other microorganisms to penetrate more easily, but also directly stimulated the bacterial growth (synergetic ability of fungal–bacterial coinfection) [31,32]. Our finding supported that antibiotics should be adjusted by the bile sampling, which may partially prevent the occurrence of IAA. Notably, one study reported that the positivity of a preoperative rectal swab had a strong correlation with biliary colonization and could also direct antibiotic prophylaxis for PD subjects without intra-operative bile sampling [33].

Our finding also demonstrated that antifungal medication should be considered in selected cases when bile yielded Candida in PD patients with clinical presentation of infection. Large-volume peritoneal lavage or prolonged use of antibiotics may be considered to decrease bacterial contamination during PD to minimize the abdominal-cavity microorganism load [34]. Sugiura et al. sampled lavage fluid after ≤7000 mL of normal-saline irrigation in PD subjects and reported that ascites contamination was associated with surgical-site infections and grades B/C pancreatic fistula [35]. Notably, 22.1% of the study subjects still had positive bacterial cultures in irrigated ascites even after extensive normal-saline irrigation of the abdominal cavity, which did not completely eradicate the pathogens. Further, Sourrouille et al. suggested extending the postoperative antimicrobial therapeutic duration to ≤5 days, because it decreased the infectious complications rate in patients at high risk for bile contamination [36]. In our institution, use of antibiotics has been extended up to postoperative day five, while positive bile cultures still indicate a risk for intra-abdominal abscess. Although antibiotics are an adjunct to both drainage and surgical debridement for controlling infectious sources to prevent ongoing major infectious complications or mortality, they may only partly attenuate abdominal-cavity contamination. A nontraumatic biliary-stump clamp and common bile-duct transection may be used during late-stage PD to lower this potentially preventable complication.

This study has the following limitations: The study design was retrospective and was conducted in a single institute; therefore, the findings may not be generalizable. Large, prospective, randomized clinical trials are warranted to validate the correlation between bile contamination and IAA; however, this is challenging to execute because we did not immediately know the results of bile cultures during PD. Second, a diverse definition of the diagnostic criteria for IAA was used in clinical research. Some studies defined IAA based on a radiologically proven collection with clinical febrile episodes. According to the American College of Surgeons National Surgical Quality Improvement Program criteria, IAA was defined based on clinical or radiological diagnosis, but the accuracy was too low in pancreatic surgery [37]. Further, there is a gray area as to the turbid fluid from drains placed during the principal operative procedure. In our study, clinically relevant POPF was included in the adjusted model to minimize the confounding effect. Third, the antibiotic regimen recommended by the Surgical Care Improvement Project for colon/abdominal surgery consists primarily of cephalosporins and ampicillin-sulbactam. This antibiotic regimen should be discontinued within 24 hours after surgery. However, current studies suggest considering prophylactic broad-spectrum antibiotics, with a longer period for pancreaticoduodenectomy patients [26,38]. In our institute, due to the complex procedures and enteric contamination, the duration of prophylactic antibiotics for PD was increased from one day to at least five days. We observed that superficial surgical site infections decreased while the IAA rate was stationary. The most effective regimen and duration of prophylactic antibiotics should be addressed in further randomized clinical trials.

## 5. Conclusions

Specific bile microorganisms, including bacteria and fungus, are the highly significant factors associated with the development of IAA. The strategy to prevent bile leakage from the biliary stump should be considered to minimize the contamination of the abdominal cavity and prevent IAA. Further, antibiotics should be adjusted according to post-operative condition and bile cultures, and anti-fungal medication may be considered in selected cases.

## Figures and Tables

**Table 1:** Comparison of the study subjects between with and without bile contamination.

Variable	Bile Contamination (*n* = 539)		
Clinical Variable	No (*n* = 106)	Yes (*n* = 433)	*p* Value
Age, y, median (IQR)	60.8 (52.5–70.2)	66.4 (57.1–74.0)	0.006
Gender			0.630
Female	46 (43.4%)	199 (46.0%)	
Male	60 (56.6%)	234 (54.0%)	
BMI, median (IQR)	22.2 (20.4–24.6)	22.5 (20.7–24.6)	0.630
Charlson comorbidity index score			0.063
≤2	27 (25.5%)	76 (17.6%)	
>2	79 (74.5%)	357 (82.4%)	
ASA physical status			0.720
<3	35 (33.0%)	151 (34.9%)	
≥3	71 (67.0%)	282 (65.1%)	
Pancreatitis history	15 (14.2%)	51 (11.8%)	0.500
Pathology			0.960
Periampullary cancer	68 (64.2%)	279 (64.4%)	
Non periampullary cancer			
Benign or low malignant neoplasm *	27 (25.5%)	100 (23.1%)	
Chronic pancreatitis	10 (9.4%)	50 (11.6%)	
Neuroendocrine tumor	1 (0.9%)	4 (0.9%)	
Preoperative T-BIL level (mg/dL, median, range)			
T-BIL Maximum at any time	0.9 (0.6–5.8)	2.6 (1.0–9.8)	<0.001
T-BIL, Maximum within 2 day before surgery	0.9 (0.6–3.2)	1.4 (0.9–2.9)	0.001
Preoperative usage of antibiotics within 30 days	53 (50.0%)	303 (70.0%)	<0.001
Preoperative biliary drainage	21 (19.8%)	181 (41.8%)	<0.001
Percutaneous transhepatic cholangiography & drainage	18 (17.0%)	39 (9.0%)	0.061
Endoscopic retrograde biliary drainage	3 (2.8%)	142 (32.8%)	<0.001
Operation time, min, median (IQR)	223.5 (194.0–268.0)	260.0 (225.0–310.0)	<0.001
Intraabdominal abscess	1 (0.9%)	74 (17.1%)	<0.001
Superficial surgical site infection	4 (3.8%)	14 (3.2%)	0.780
Clinically relevant POPF	28 (26.4%)	110 (25.4%)	0.610

BMI, Body Mass Index; ASA physical status, American Society of Anesthesiologists physical status; T-BIL, Total bilirubin; POPF postoperative pancreatic fistula. * Mucinous or serous cystadenoma, intraductal papillary mucinous neoplasm, solid pseudopapillary epithelial neoplasm.

**Table 2:** Statistical analysis of microorganisms to predict intra-abdominal abscess.

Microorganisms	Odds Ratio	95% CI	*p* Value
Enterococcus species	4.156	2.661–6.491	<0.001
Enterobacter species	2.515	1.546–4.093	<0.001
Escherichia Coli	2.333	1.384–3.933	0.001
Klebsiella species	1.195	0.697–2.048	0.517
Streptococcus species	2.796	1.502–5.206	0.001
Citrobacter species	3.376	1.686–6.762	0.001
Pseudomonas Aeruginosa	1.783	0.889–3.581	0.104
Aeromonas species	2.094	1.002–4.376	0.057
Clostridium species	0.728	0.316–1.676	0.455
Proteus species	1.099	0.469–2.573	0.827
Candida species	4.994	1.837–13.572	0.002

**Table 3:** Univariate analysis and adjusted multivariate analysis to predict intra-abdominal abscess.

Clinical Variant	Univariate *p* Value	Multivariate		
		Odds Ratio	95% CI	*p* Value
Age	<0.001	1.023	1.004–1.041	0.014
Male	0.580	1.151	0.769–1.724	0.493
ASA ≥ 3	0.990	0.862	0.544–1.366	0.529
BMI	0.240	1.005	0.946–1.067	0.867
T-BIL, Maximum within 2 days before surgery	0.140	0.969	0.905–1.039	0.386
Antibiotic use within 30 days before surgery	0.170	0.930	0.598–1.447	0.750
Preoperative biliary drainage	0.010			
Percutaneous transhepatic cholangiography and drainage (ref: no PBD)	0.869	1.001	0.461–2.171	0.997
Endoscopic retrograde biliary drainage (ref: no PBD)	<0.001	1.176	0.676–2.047	0.565
CCI score > 2	0.105	1.610	0.812–3.193	0.172
Periampullary cancer	0.020	1.041	0.626–1.731	0.876
Pancreatitis	0.510	1.067	0.553–2.056	0.846
Clinically relevant postoperative pancreatic fistula	0.610	0.882	0.591–1.316	0.539
Microorganism				
Candida species	0.002	4.666	1.677–12.981	0.003
Enterococcus species	< 0.001	4.103	2.609–6.454	<0.001
Citrobacter species	0.001	3.050	1.510–6.159	0.002
Enterobacter species	<0.001	2.231	1.349–3.689	0.002
Streptococcus species	0.001	2.519	1.331–4.767	0.005
Escherichia Coli	0.001	2.215	1.336–3.671	0.002

BMI, Body Mass Index; ASA physical status, American Society of Anesthesiologists physical status; T-BIL, Total-bilirubin; CCI score, Charlson comorbidity index score, POPF postoperative pancreatic fistula.

## Data Availability

The data presented in this study are available on request from the corresponding author.

## Supplementary Materials

The following are available online at https://www.mdpi.com/article/10.3390/curroncol29010009/s1, Table S1: Adjusted multivariate analysis to predict intra-abdominal abscess by including ineffective therapy as a variable.
