# Supplementary material for: Specific Bile Microorganisms Caused by Intra-Abdominal Abscess on Pancreaticoduodenectomy Patients: A Retrospective Cohort Study
Source: Curr Oncol. 2021 Dec 27;29(1):111–21. doi: 10.3390/curroncol29010009 (PMC8774444; doi:10.3390/curroncol29010009)
Supplement: Supplementary file 1 [file curroncol-29-00009-s001.zip › curroncol-1471748-supplementary .pdf]

Current Oncology

Table S1. Adjusted multivariate analysis to predict intra-abdominal abscess by including ineffective therapy as a variable.

Clinical Variant	Odds Ratio	95% CI	*p* Value
Age	1.023	1.006–1.040	0.007
Male	1.031	0.709–1.500	0.870
ASA ≥ 3	0.785	0.515–1.199	0.264
BMI	1.023	0.968–1.080	0.414
T-BIL Maximum at any time	1.001	0.959–1.044	0.963
T-BIL, Maximum within 2 day before surgery	0.940	0.885–1.009	0.095
Antibiotic use within 30 day before surgery	1.011	0.674–1.517	0.955
Preoperative biliary drainage	1.461	0.905–2.357	0.120
CCI score > 2	1.626	0.872–3.032	0.126
Periampullary cancer	1.145	0.714–1.835	0.573
Pancreatitis	1.115	0.607–2.048	0.724
Pancreatic fistula	0.806	0.555–1.170	0.257
Ineffective therapy	2.727	1.840–4.040	<0.001

BMI, Body Mass Index; ASA physical status, American Society of Anesthesiologists physical status; T-BIL, Total-bilirubin; CCI score, Charlson comorbidity index score.
